# Time-Dependent Degradation Mechanism of Basalt Fiber Reinforced Polymer (BFRP) in a Low-Alkalinity Environment

**DOI:** 10.3390/ma18174170

**Published:** 2025-09-05

**Authors:** Weiwen Li, Murong Zou, Meilin He, Wanye Li, Peng Wang, Yihong Tang

**Affiliations:** 1 Guangdong Provincial Key Laboratory of Durability for Marine Civil Engineering, Shenzhen University, Shenzhen 518060, China; 2 Department of Civil and Environmental Engineering, The Hong Kong University of Science and Technology, Hong Kong, China

**Keywords:** basalt fiber reinforced polymer (BFRP), durability, exposure pH, diffusion coefficient, microscopic characteristics

## Abstract

Basalt fiber reinforced polymer (BFRP) has been utilized as a corrosion-resistant substitute for steel rebar in concrete structures. However, embedded BFRP rebars may degrade over time within the alkaline concrete pore solution. While extensive literature has scrutinized BFRP degradation under highly alkaline conditions (e.g., pH~13 in normal concrete), comparatively few studies have addressed its behavior under lower alkalinity (e.g., pH~11–12 in carbonated/green concrete). To address this issue, this study systematically investigates the degradation mechanism of BFRP rebars under coupled factors of pH (7, 11, 12, and 13), temperature (23, 40, and 60 °C), and aging time (30, 60, and 90 days). Research outcomes indicate that a decrease in pH from 13 to 11 at 23 °C results in a reduction in diffusion coefficient from 7.071 × 10^−7^ mm^2^/s to 5.876 × 10^−7^ mm^2^/s. Moreover, lowering the temperature from 60 °C to 23 °C at pH 12 leads to a decline in the diffusion coefficient from 7.547 × 10^−7^ mm^2^/s to 6.758 × 10^−7^ mm^2^/s. Furthermore, following a 90-day immersion at 60 °C, decreasing the exposure pH from 13 to 11 can significantly improve tensile strength retention from 25.357% to 71.933%. In the same scenario, flexural strength retention (or interlaminar shear strength retention) increases from 20.930% to 87.638% (or 23.464% to 76.592%) in such a mildly alkaline environment. A comprehensive degradation mechanism is uncovered, linking macroscopic mechanical properties to microscopic characteristics (encompassing fiber corrosion, matrix cracking, and interfacial debonding). This degradation process can be expedited by alkali attack and thermal activation. These findings contribute valuable insights into the alkali-induced degradation process and furnish a comprehensive dataset regarding the durability performance of BFRP rebars.

## 1. Introduction

With the ongoing progression of infrastructure development, the importance of structural durability is gaining recognition, primarily due to the susceptibility of conventional steel rebar to corrosion [1]. Basalt fiber reinforced polymer (BFRP) has emerged as a promising construction material, characterized by its lightweight nature, superior strength, and excellent corrosion resistance [2,3,4]. These attributes make BFRP a highly promising alternative to steel, effectively addressing the structural durability challenges associated with steel corrosion [5]. Nonetheless, the long-term performance of BFRP under alkaline attack remains insufficiently understood, particularly in low-alkalinity environments such as carbonated or green concrete. In these scenarios, the cement content is often substituted with calcium carbonate (CaCO_3_) or supplementary cementitious materials (SCMs) such as fly ash and silica fume, leading to pH levels typically ranging from 11 to 12. Such low-alkalinity conditions may be adverse to embedded steel rebars but facilitate the durable performance of BFRP rebar compared to the normal concrete (pH~13). Given the substantial engineering potential of carbonated and green concrete in conjunction with BFRP rebars, it is essential to conduct a comprehensive investigation into the durability performance of BFRP rebars in low-alkalinity environments.

Numerous scholars and engineers have confirmed BFRP degradation phenomena in bench-scale studies and practical engineering applications. The presence of an alkaline medium can erode basalt fibers [6] and the polymer matrix [7], as well as disrupt the fiber-matrix interface [8,9,10], thereby resulting in a significant loss in the mechanical properties of BFRP rebars [11] (including tensile strength [12,13,14], flexural strength, and interlaminar shear strength [15]). Notably, temperature serves as a critical accelerator in this BFRP degradation process. For every 10 °C increase, the time required to achieve equivalent strength loss is reduced by 1.5 to 2 times, owing to enhanced chemical reactivity [9]. Actually, throughout the service life of BFRP rebars, they are subjected to the combined influences of alkalinity and temperature, which synergistically accelerate material degradation and shorten structural service life. However, most existing studies primarily focus on high-alkalinity scenarios, leaving a critical knowledge gap in understanding BFRP degradation behavior in low-alkalinity environments. Therefore, a quantitative assessment of BFRP degradation in low-alkalinity concrete is essential for advancing durability design and extending the service life of BFRP-reinforced concrete structures.

Although multi-factor durability studies have addressed both GFRP and BFRP reinforcing bars, the literature remains more comprehensive for GFRP, with relatively limited assessments of BFRP under low-alkalinity conditions. Wang et al. [16,17,18] comprehensively investigated GFRP rebars across reduced pH (13 to 7) and temperature (60 °C to 23 °C), demonstrating that lower alkalinity substantially mitigates alkali-induced fiber/matrix defects and tensile strength loss, while exerting minimal influence on flexural and interlaminar shear strengths. Furthermore, Fergani et al. [19] evaluated GFRP degradation under combined environmental exposure and sustained loading, underscoring the role of stress-assisted damage in long-term performance.

For BFRP, prior work has usually examined individual or paired stressors, with limited systematic exploration of synergistic effects among alkalinity, temperature, and aging time, particularly with quantitative treatment of diffusion and mechanical degradation. Altalmas et al. [20] reported that an alkaline solution at pH 12.5 reduced BFRP bond between BFRP bars and concrete strength by 25% after 90 days, with interfacial failure characterized by interlaminar shear damage, owing to chemical erosion at the fiber-matrix interface due to alkali attack. Similarly, Wang et al. [21] observed faster strength loss over time for BFRP in pH 13.4 solution, with higher activation energy at elevated pH. In addition, Chen et al. [22] showed that temperature–pH synergy markedly accelerates creep-fracture deterioration in BFRP.

Despite these contributions, systematic and quantitative evaluations of BFRP in low-alkalinity environments remain scarce. This narrow focus often overlooks critical degradation modes, such as reduced tensile strength, decreased interlaminar shear strength, and deteriorated bending performance, and coupled damage mechanisms (e.g., interfacial debonding, matrix cracking, and fiber erosion) that arise under more realistic low-alkalinity conditions involving multiple environmental factors (e.g., pH 11–12, moisture, and temperature) [23,24]. Addressing this gap necessitates systematic experimental studies to assess the coupled effects of these variables in low-alkalinity environments. Such insights are essential for the reliable engineering application of BFRP in moderately alkaline corrosive settings, including carbonated or green concrete structures with much lower alkalinity [25,26].

To address this gap, the present study thoroughly investigates the evolution of mechanical properties in BFRP rebars under the coupled effects of low alkalinity, temperature, and aging time. Controlled immersion tests are performed at pH 11 and 12, with pH 7 as a reference and pH 13 for comparison, at temperatures of 23 °C, 40 °C, and 60 °C. The experiments systematically characterize water absorption and mechanical performance under low-alkalinity conditions, quantify the role of moisture ingress in material deterioration, and elucidate degradation mechanisms arising from the multi-factor interaction of alkalinity, temperature, and exposure duration. The results provide a basis for assessing long-term performance and informing the rational use of BFRP rebars in low-alkaline engineering environments.

## 2. Experimental Program

### 2.1. Materials and Specimens

Physical and mechanical properties of pultruded BFRP rebars are tabulated in Table 1. Also, the chemical composition of BFRP is analyzed using X-ray fluorescence (XRF) and presented in Table 2. These BFRP rebars possess a fiber volume fraction of approximately 60%, facilitating effective load sharing among fibers and efficient load transfer between basalt fibers and the surrounding matrix. The porosity is approximately 2%, which can be attributed to high-quality polymer impregnation and a tightly controlled pultrusion process during manufacturing.

**Figure 1:**
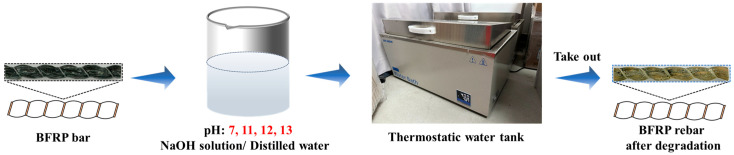
Bench-scale short-term acceleration test of BFRP rebar.

As shown in Figure 1, to establish a stable alkaline environment, solutions are prepared with NaOH to achieve accurate and consistent pH levels. Four solutions with distinct alkalinity (pH 7, 11, 12, and 13) are obtained by dissolving sodium hydroxide (NaOH) in distilled water. To minimize alkalinity loss due to reactions between NaOH and atmospheric carbon dioxide (CO_2_), all solutions are replaced monthly throughout the aging period. For each pH condition, three temperatures (23 °C, 40 °C, and 60 °C) are maintained by controlling the water bath temperature.

BFRP rebars are extracted from the solutions for mechanical tests only after 30, 60, and 90 days of immersion. All these tests are destructive; specimens are not reintroduced into the solutions. The test program comprises uniaxial tensile, three-point bending, and interlaminar shear tests to determine mechanical properties (e.g., tensile strength $\sigma_{TSL}$, flexural strength $\sigma_{FLX}$, and interlaminar strength $\tau_{ILSS}$) and to evaluate degradation over predefined aging intervals.

For the water absorption test, BFRP rebars are temporarily taken out, tested, and then returned to the solution for continued immersion, enabling non-destructive assessment. The tested BFRP rebars are weighed after a predefined aging time of up to 18 months of immersion. After each weighing for the water absorption test, the rebars are immediately placed back into their original immersion containers to ensure they remain continuously exposed to the predefined environmental conditions without interruption.

For water absorption tests, BFRP rebars are immersed in dedicated containers, with each container labeled clearly on its exterior to indicate the solution’s pH, temperature, and immersion start date (e.g., “pH 13, 60 °C, Start: 1 January 2024”). Inside each container, individual rebars are numbered sequentially to track replicates. These numbers remain consistent as the rebars are temporarily removed for weighing and promptly returned to the same container to maintain continuous exposure. Upon extraction for testing at 30, 60, or 90 days, each rebar is assigned a unique identifier that encodes the solution pH, temperature, exposure duration, test type, and specimen number (e.g., “BFRP-13-60-90d-T-1” denotes the first BFRP rebar immersed in pH 13 at 60 °C and tested in tension after 90 days; “BFRP-11-40-60d-F-2” denotes the second specimen subjected to flexural testing after 60 days in pH 11 at 40 °C). These tests are destructive, so specimens are not reintroduced into the solutions after testing. The labeling ensures clear traceability of each specimen’s exposure history and test purpose.

### 2.2. Water Absorption Test

During the water absorption test, the pre-cut 60-mm BFRP rebars are removed from the solutions. For the water absorption test, after removing BFRP specimens from the alkaline solution at each scheduled aging interval, surface moisture is carefully wiped off using towel tissue to achieve a surface-dry state. After that, the BFRP rebars are weighed on a high-precision electronic scale, and the data are meticulously recorded. The water absorption of BFRP rebars can be calculated as follows:

(1) $$M_{t}\;=\;\frac{M_{w}\;-\;M_{0}}{M_{0}}\;\times \;100\%$$

where $M_{0}$ is the initial BFRP rebar weight and $M_{w}$ is the soaked BFRP rebar weight.

The diffusion coefficient D is determined using Equation (2). In the initial stage of absorption, the uptake $M_{t}$ is approximately proportional to the square root of immersion time $t^{\frac{1}{2}}$ [30]. $M_{\infty }$ is the maximum water uptake at saturation. Accordingly, D can be derived from the slope of the linear fit to the early-time plot of water absorption versus $t^{\frac{1}{2}}$.

(2) $$\frac{M_{t}}{M_{\infty }}={4\left(\frac{Dt}{\pi a^{2}}\right)}^{\frac{1}{2}}-\frac{Dt}{a^{2}}-{\frac{\pi }{3}\left(\frac{Dt}{\pi a^{2}}\right)}^{\frac{3}{2}}+\cdots \;\approx \;{4\left(\frac{Dt}{\pi a^{2}}\right)}^{\frac{1}{2}}$$

### 2.3. Mechanical Test

**Figure 2:**
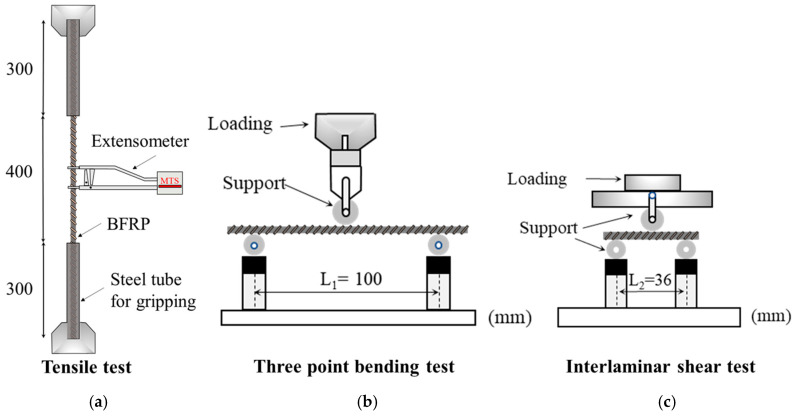
Mechanical testing device: (**a**) tensile test, (**b**) three-point bending test, (**c**) interlaminar shear test.

*Tensile test:* As per ASTM D7205M-06 [27], both ends of BFRP rebar are placed within 300-mm-long steel tubes, and the hollow space between BFRP rebar and steel tube is filled with epoxy resin (Sika 330, produced by Guangzhou Sika Building Materials Co., Ltd., Guangzhou, China) to ensure sufficient load transfer during the tensile test. Steel tubes with BFRP rebar are then stretched by MTS wedges at a rate of 0.015 mm/s. A 50-mm extensometer is employed to measure strain in BFRP rebar and to calculate the elastic modulus (Figure 2a [24]). Experimental data are automatically recorded by the computational acquisition system at 0.05 s intervals. Three identical specimens are tested to determine the average tensile strength of BFRP rebars. The tensile strength $\sigma_{TSL}$ and corresponding elastic modulus $E_{TSL}$ are calculated as follows:

(3) $$\sigma_{TSL}\;=\;\frac{P}{\pi a^{2}}$$

(4) $$E_{TSL}=\frac{\sigma_{2}-\sigma_{1}}{\varepsilon_{2}-\varepsilon_{1}}$$

where P is the ultimate tensile force (N), a is the nominal radius (mm) of BFRP rebar, $\sigma_{1}$, $\sigma_{2}$, $\varepsilon_{1}$, and $\varepsilon_{2}$ represent the tensile stress and corresponding strain at two points on the initial linear stage of the σ-ε curve.

*Three-point bending test:* In accordance with ASTM D4476-14 [28], 120-mm BFRP rebars are employed for the three-point bending test herein. Two rigid support rollers are spaced at $L_{1}$ = 100 mm, and a loading roller is applied at midspan (Figure 2b [24]). Loading is performed using an electromechanical test machine (ETM) (ETM305D, produced by Wance Co., Ltd., Shenzhen, China) with a 250 kN capacity at a displacement rate of 3 mm/min. Three identical specimens are tested to determine the average flexural strength $\sigma_{FLX}$ and corresponding elastic modulus $E_{FLX}$ of BFRP rebars:

(5) $$\sigma_{FLX}\;=\;\frac{P_{1}L_{1}}{\pi a^{3}}$$

(6) $$E_{FLX}=\frac{∆P_{1}L_{1}^{3}}{12\pi a^{4}\cdot ∆L_{1}}$$

where $P_{1}$ is the ultimate flexural force (N), $∆P_{1}$ and $∆L_{1}$ represent the difference in force and displacement between two points on the linear stage of the force-displacement curve.

*Interlaminar shear test:* The shear resistance at the fiber-matrix interface of BFRP rebar is measured in compliance with ASTM D4475-02 [29]. These BFRP rebars are 42 mm in length, with the span between the two rigid support rollers set to $L_{2}$ = 36 mm (Figure 2c [24]). A loading roller is applied at midspan. Loading is conducted using an EZ50 testing machine (Lloyd, New York, NY, USA) with a 30 kN capacity at a rate of 1.3 mm/min. Three identical specimens are tested to determine the average interlaminar shear strength $\tau_{ILSS}$ and corresponding elastic modulus $G_{ILSS}$ of BFRP rebars:

(7) $$\tau_{ILSS}\;=\;\frac{0.212P_{2}}{\pi a^{2}}$$

(8) $$G_{ILSS}=\frac{0.106\cdot ∆P_{2}\cdot L_{2}}{\pi a^{2}\cdot ∆L_{2}}$$

where $P_{2}$ is the ultimate interlaminar shear force (N), $∆P_{2}$ and $∆L_{2}$ represent the difference in force and displacement, respectively, between two points on the linear stage of the force-displacement curve.

### 2.4. Microstructural Analysis

*Scanning electron microscopy (SEM):* Cross-sections of BFRP rebars are prepared using a diamond saw. The cut specimens are then polished with SiC papers of 600, 800, and 1200 grit at a rotational speed of 100 rad/s. Afterwards, the specimens are mounted on a copper stage with carbon tape, with the polished surface oriented upward. To improve surface conductivity, a thin gold film is sputter-coated onto the specimens. Microstructural characterizations are performed using an SEM (Quanta TM 250 FEG, Raleigh, NC, USA).

*Differential scanning calorimetry (DSC):* The thermal behavior of BFRP rebars is characterized herein. A 1.5 mg specimen is precision-cut from the rebar surface using a diamond saw. Measurements are conducted on a TA instrument (New Castle, DE, USA) (Q1000) in a nitrogen atmosphere to prevent oxidation during the heating process. The temperature increases from room temperature (23 °C) to 200 °C at a rate of 10 °C/min. Throughout the thermal program, the rebar exhibits a glass-to-rubber transition, identified as the glass transition temperature ($T_{g}$). A two-cycle heating protocol is employed to ensure measurement reliability: the first cycle achieves complete matrix polymerization, while the second heating cycle provides the definitive $T_{g}$, reflecting the intrinsic thermal transition of virgin and conditioned BFRP rebars.

*Fourier transform infrared spectroscopy (FTIR):* A sliced BFRP specimen is ground to a fine powder and sieved to 200 μm. Functional groups and associated chemical bonds are identified to elucidate potential reactions under various exposure conditions. The powder is analyzed in the mid-infrared region (400–4000 cm^−1^) using a Vertex 70 Hyperion 1000 (Bruker, Billerica, MA, USA) FTIR spectrometer. A total of 64 scans are acquired at a resolution of 4 cm^−1^. The background spectrum is subtracted prior to analysis to minimize environmental interference and enhance the accuracy of functional group bond identification.

## 3. Results

### 3.1. Surface Appearance Observation

**Figure 3:**
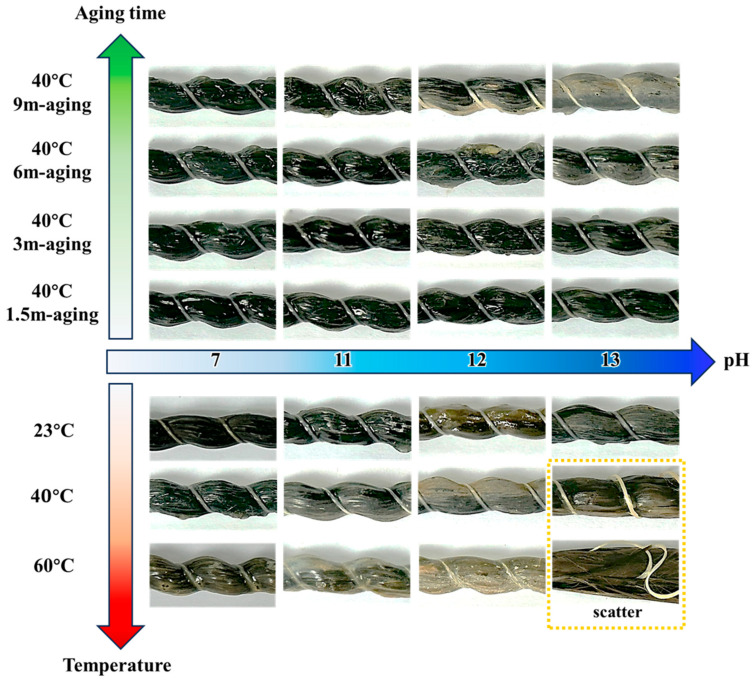
Appearance of BFRP rebars under distinct environmental conditions.

Figure 3 illustrates the synergistic influence of exposure pH and ambient temperature on the surface morphology of conditioned BFRP over time. Alkaline environments (pH 7, 11, 12, and 13) progressively degrade the rebar surfaces, with damage severity increasing with both pH, temperature, and exposure duration. In particular, under the most aggressive condition (i.e., pH 13 at 60 °C), the BFRP rebars exhibit pronounced fiber bundle dispersion and structural disintegration following prolonged exposure.

Exposure pH plays a critical role in BFRP degradation. Distilled water (pH 7) causes minimal damage, whereas NaOH solutions at moderate alkalinity (pH 11–12) induce surface roughening. When the BFRP rebars are immersed in distilled water (pH 7), their surfaces remain largely smooth after 9-month exposure at 60 °C, showing only minor discoloration or slight roughness. Increasing alkalinity to pH 11 leads to localized surface roughening after 3 months at both 40 °C and 60 °C.

Temperature also exerts a significant influence on BFRP degradation. Under the most aggressive condition (pH 13 and 60 °C), fiber bundle separation is triggered, whereas lower temperatures primarily limit damage to surface softening. For instance, the specimens at pH 13–60 °C undergo a markedly accelerated degradation, with fiber bundles loosened, separated, and dispersed from the matrix, resulting in structural disintegration. By contrast, regarding specimens at pH 13–23 °C and pH 13–40 °C, damage is largely confined to surface softening, without obvious fiber bundle scattering even after 9-month exposure.

### 3.2. Water Absorption Results

**Figure 4:**
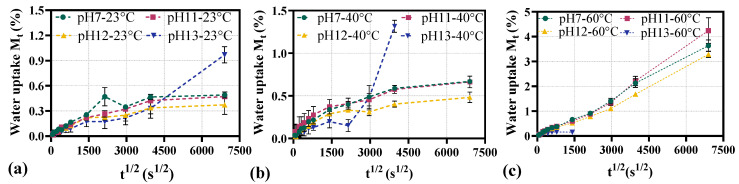
Water absorption of BFRP rebars: (**a**) 23 °C; (**b**) 40 °C; (**c**) 60 °C.

Figure 4 illustrates the variation of water uptake $M_{t}$ with the square root of time $t^{\frac{1}{2}}$ for BFRP rebars exposed to different pH values (7, 11, 12, and 13) and temperatures (23 °C, 40 °C, and 60 °C). In Figure 4a, the absorption curves for all pH conditions exhibit a two-stage water absorption pattern when the ambient temperature is 23 °C [30]. In the initial stage (approximately corresponding to $t^{\frac{1}{2}}$ < 500$\;s^{\frac{1}{2}}$), $M_{t}$ increases linearly with $t^{\frac{1}{2}}$, consistent with the Fick’s model [31]. As $t^{\frac{1}{2}}$ further increases, the curves of pH 7, pH 11, and pH 12 gradually become flat, indicating the full saturation of BFRP rebar. In contrast, the curve for pH 13 continues to rise more noticeably. A similar two-stage behavior is observed for BFRP rebars at 40 °C in Figure 4b, likely attributable to progressive microstructural damage that generates additional micro voids, facilitating further water uptake.

For BFRP rebars in 60 °C solution in Figure 4c, all curves exhibit an initial linear increase in $M_{t}$ with $t^{\frac{1}{2}}$, following Fick’s model. With prolonged exposure, however, the trajectories deviate upward rather than approaching a plateau. This deviation likely arises from temperature-accelerated chemical degradation of BFRP rebar, indicating that transport is no longer purely physical; degradation-induced microstructural evolution increases the material’s water uptake capacity.

All diffusion coefficient data are computed and tabulated in Table 3. It can be seen that pH and temperature have a significant effect on the diffusion behavior [32]. For example, the diffusion coefficient increases from 5.876 × 10^−7^ mm^2^/s in distilled water (pH = 7) to 7.071 × 10^−7^ mm^2^/s in sodium hydroxide solution (pH = 13) at room temperature (23 °C), indicating the higher alkaline environment accelerates water diffusion. Moreover, elevated temperature accelerates the diffusion, as evidenced by the increase of the diffusion coefficient from 5.876 × 10^−7^ mm^2^/s at 23 °C to 7.849 × 10^−7^ mm^2^/s at 60 °C in distilled water (pH = 7).

It should be noted that for the case of pH 13-60 °C, the BFRP rebars undergo extensive degradation, manifested by dispersed fiber bundles and loss of structural integrity observable at the macroscopic surface in Figure 3, rendering reliable diffusion measurements infeasible in Table 3.

### 3.3. Degradation of Mechanical Properties

#### 3.3.1. Tensile Strength Retention

**Figure 5:**
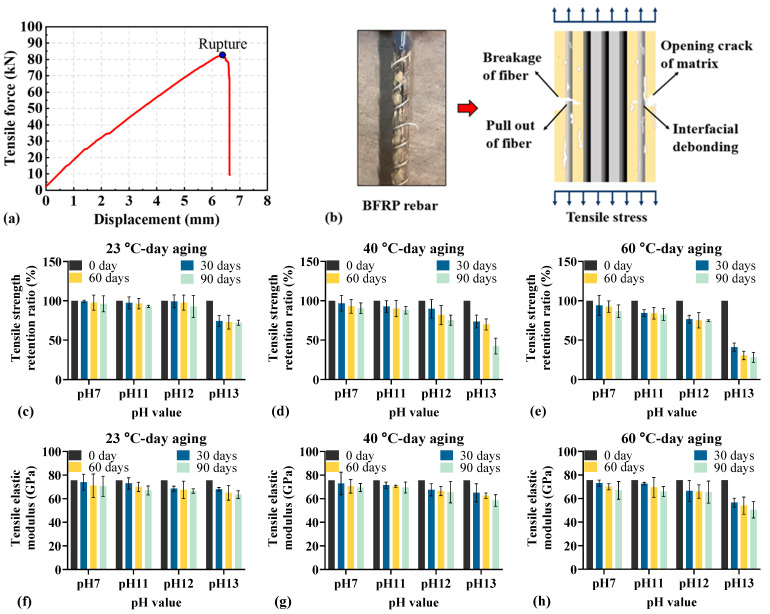
Tensile behavior of BFRP rebars under different environmental conditions: (**a**) typical force-displacement curve; (**b**) stress distribution and failure mode; (**c**–**e**) tensile strength retention; (**f**–**h**) elastic modulus.

Figure 5 illustrates the tensile strength retention ratio and elastic modulus of BFRP rebar under different environmental conditions. As shown in Figure 5a,b, the tensile force increases linearly in the initial stage. Upon increasing tensile force, microcracks develop within the polymer matrix, accompanied by the interfacial debonding between the matrix and fibers. Finally, the tensile force decreases suddenly with the BFRP rebar’s rupture.

As illustrated in Figure 5c–e, the tensile strength retention ratio shows a downward trend with the prolonged aging time. At a fixed pH, higher temperatures lead to more rapid and pronounced performance degradation. For example, at pH 12, increasing the temperature from 40 °C to 60 °C reduces the time to reach a comparable tensile strength retention (24.656% at 40 °C vs. 23.398% at 60 °C) from 90 days to 30 days. Furthermore, increasing pH exacerbates the decline in tensile strength retention. In particular, at pH 13 the reduction is substantially greater than at pH 11 or 12, ranging from 25.680% to 71.933% at pH 13, compared with 2.568% to 17.354% at pH 11 and 0.726% to 25.357% at pH 12, indicating that stronger alkalinity imposes a more severe detrimental effect on the tensile performance of BFRP rebars.

Figure 5f–h show that, at fixed pH and aging time across different temperatures, the tensile elastic modulus of conditioned BFRP rebars decreases less than the tensile strength. This suggests that, when other environmental factors are controlled, temperature exerts only a minor influence on the tensile elastic modulus of BFRP rebars.

#### 3.3.2. Flexural Strength Retention

**Figure 6:**
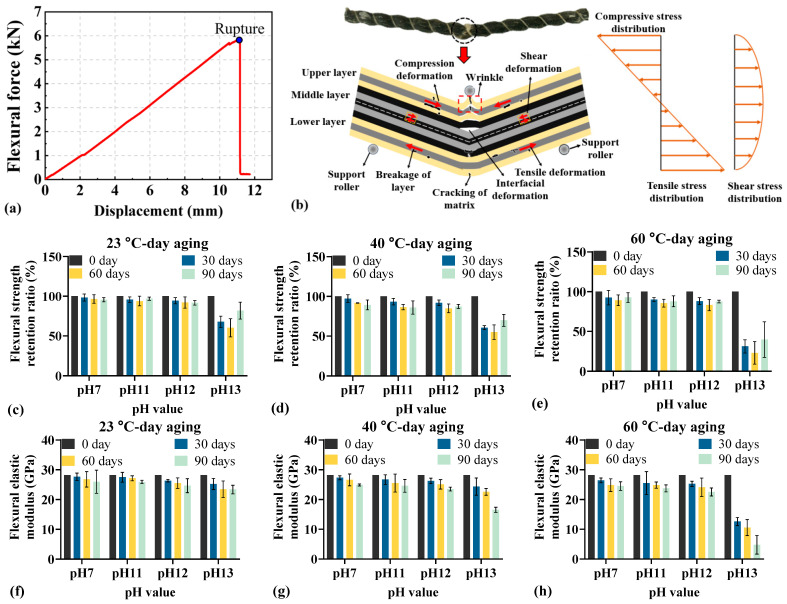
Flexural behavior of BFRP rebars under different environmental conditions: (**a**) typical force-displacement curve; (**b**) stress distribution and failure mode; (**c**–**e**) flexural strength retention; (**f**–**h**) elastic modulus.

Figure 6 presents the flexural behavior of BFRP rebars under different environmental conditions. Figure 6a shows the typical force-displacement curve of BFRP rebar in the flexural test, where the bending force increases nearly linearly with axial displacement until rupture occurs. As shown in Figure 6b, flexural loading induces compression and wrinkling in the upper layer of the BFRP rebar; interfacial deformation and matrix cracking in the mid-layer; and tensile deformation, laminar fracture, and shear deformation in the lower, tensioned layer. Ultimately, bending failure occurs, governed primarily by damage in the lower tension zone, with contributory effects from compressive damage in the upper layer and interfacial deformation in the mid-layer.

As shown in Figure 6c–e, flexural strength retention declines with increasing aging time. At a fixed pH, higher temperatures accelerate and intensify the degradation. For instance, at pH 13 the reduction in flexural strength is greater at 60 °C than at 23 °C or 40 °C (87.638% at 60 °C vs. 44.495% at 23 °C and 57.744% at 40 °C). Moreover, increasing pH further amplifies the loss: at 60 °C, the decrease at pH 13 (87.638%) substantially exceeds that at pH 11 (20.930%) and pH 12 (23.073%), indicating that strong alkalinity more severely compromises the flexural strength of BFRP rebars.

Figure 6f–h indicate that the flexural elastic modulus of BFRP rebars decreases with aging under fixed pH and varying temperatures. In the case of pH 7–23 °C (Figure 6f), the modulus declines from 28.20 GPa to 25.98 GPa (−2.22 GPa). Under more severe conditions of pH 11–40 °C (Figure 6g), it decreases from 28.20 GPa to 24.59 GPa (−3.61 GPa). The most pronounced loss occurs in the case of pH 13–60 °C (Figure 6h), where the modulus drops from 28.20 GPa to 4.79 GPa (−23.41 GPa). Consistently, as shown in Figure 6c–e, at 60 °C and pH 13, the flexural strength retention declines from 100% at day 0 to 12.367% at day 90 (−87.632%). Thus, both flexural elastic modulus and strength retention deteriorate substantially under harsh conditions.

#### 3.3.3. Interlaminar Shear Strength Retention

**Figure 7:**
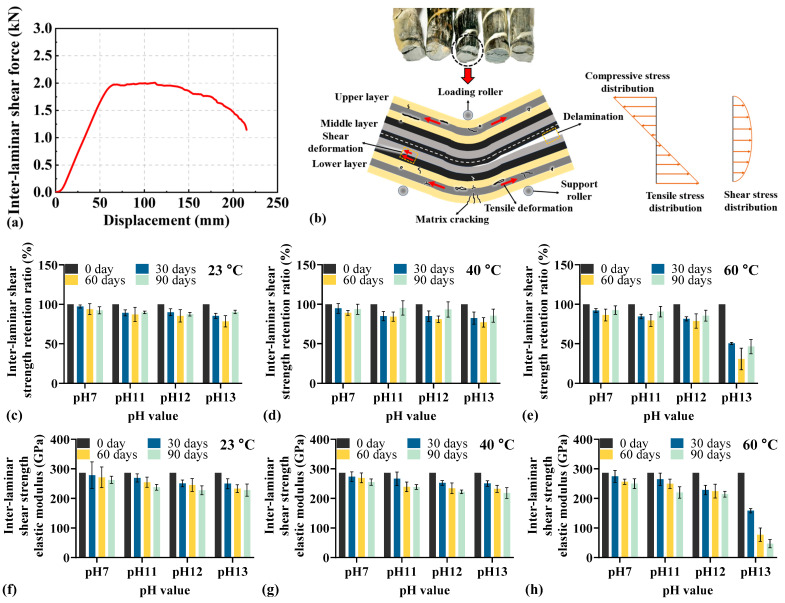
Interlaminar shear behavior of BFRP rebars under different environmental conditions: (**a**) typical force-displacement curve; (**b**) stress distribution and failure mode; (**c**–**e**) interlaminar shear strength retention; (**f**–**h**) elastic modulus.

Figure 7 illustrates the interlaminar shear strength of BFRP under different environmental conditions. The interlayer shear-displacement curve in Figure 7a first rises linearly and then enters a plateau and finally decreases until rupture. The schematic diagram in Figure 7b shows that, during loading, accompanied by shear deformation, matrix cracking, and interlayer delamination, the distribution of stress on the right side indicates that shear stress concentrates on the interlayer interfaces, making these interfaces weak zones prone to debonding and delamination.

Figure 7c–e illustrate that interlaminar shear strength retention of BFRP rebar degrades with aging time, temperature, and pH. Over 30, 60, and 90 days, the retention ratio consistently declines. At a fixed pH, higher temperatures accelerate the loss. For example, when the BFRP rebar is immersed in the highly alkaline solution (pH 13), the strength reduction increases from 22.734% (23 °C) to 29.408% (40 °C) and 76.557% (60 °C). At fixed temperature, higher alkalinity further reduces retention. For instance, when the ambient temperature is 60 °C, the strength reduction increases from 14.776% (pH 7), 23.360% (pH 11), 30.168% (pH 12), and 76.557% (pH 13). Figure 7f–h also document reductions in the interlaminar shear elastic modulus with aging. In the case of pH 13-60 °C, the elastic modulus decreases by 239.376 GPa over 90 days. Overall, the elastic modulus exhibits a degradation trend consistent with that of interlaminar shear strength across temperatures and pH levels.

### 3.4. Microstructure Observation

#### 3.4.1. DSC

In engineering applications, temperature affects the modulus-related behavior of both fibers and matrix. Maintaining a stable glass transition temperature ($T_{g}$) is essential to ensure that the modulus does not degrade under service conditions, thereby preserving structural integrity and load-bearing capacity

**Figure 8:**
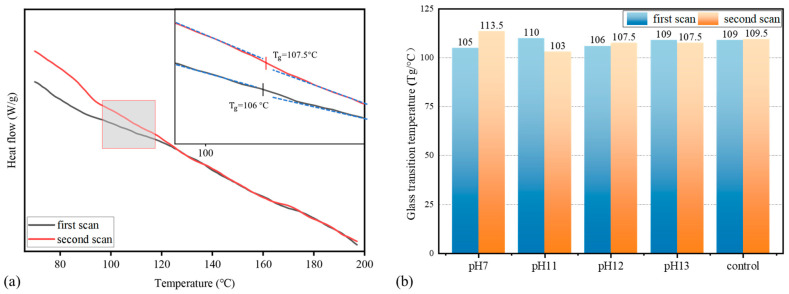
Glass transition temperature of BFRP rebar: (**a**) DSC curve after immersion in pH 12 solution at 60 °C for 12 months; (**b**) $T_{g}$ for different pH cases at 60 °C for 12 months.

Figure 8a presents a representative DSC curve of BFRP rebar after 12-month immersion in a pH 12 solution at 60 °C. Within the gray shaded zoom-in region, the heat-flow trace exhibits two distinct slopes with respect to temperature over approximately 106.0–107.5 °C. This change corresponds to the glass transition, with the inflection point ($T_{g}$) marking the temperature at which the resin matrix transitions from a glassy to a rubbery state. Figure 8b compiles the $T_{g}$ values of specimens aged at 60 °C across various pH environments for up to 12 months. $T_{g}$ values of all aged specimens are comparable to that of the control, indicating that prolonged alkaline exposure at elevated temperature does not substantially affect the resin matrix’s thermal properties. Furthermore, the measured $T_{g}$ range (106.0–113.5 °C) is well above the exposure temperature of 60 °C, implying that the matrix remains in the glassy state throughout service. In this state, polymer chain mobility is constrained, and intermolecular interactions remain stable, providing a consistent mechanical framework.

#### 3.4.2. FTIR

**Figure 9:**
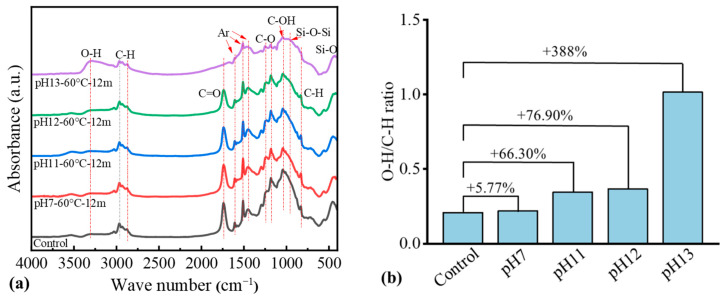
FTIR spectrum of control and conditioned BFRP rebars: (**a**) FTIR curve; (**b**) O-H/C-H ratio.

As shown in Figure 9a, using the C–H band (2800–3000 cm^−1^) as reveals the following trends: (1) The O–H stretching band (~3500 cm^−1^) increases progressively with pH, with the most pronounced growth observed in pH 13-60 °C-12 m. (2) The C=O stretching band (~1730 cm^−1^) diminishes markedly as pH increases, with the greatest attenuation in pH 13-60 °C-12 m. (3) The C–O–C ether region (1000–1250 cm^−1^) shows a gradual decline in intensity with increasing alkalinity. For pH 13-60 °C-12 m, substantial peak broadening and distortion are evident, indicating potential resin matrix degradation and disruption of the Si-O network in basalt fibers. (4) The C–OH band (1050–1150 cm^−1^) exhibits altered peak morphology and a relative decrease in intensity in aged specimens. (5) The Si–O–Si and Si–O bands (500–1000 cm^−1^) undergo peak-shape changes and intensity reductions in pH 13-60 °C-12 m.

Quantitative analysis in Table 4 further supports these trends. Compared to the control group, pH 13-60 °C-12 m exhibits an increase of 388% in the O-H/C-H ratio. Meanwhile, the C=O/C-H ratio shows a reduction of 70.4% when comparing the control group and the specimen pH 13-60 °C-12 m. This proves that the ester bond in the resin matrix is seriously hydrolyzed, and about 70% of the ester bond structure is destroyed. The C-O-C/C-H ratio decreases by 50.2%, from 4.364 in the control group to 2.173 in pH 13-60 °C-12 m, indicating severe degradation of the polymetric three-dimensional network structure. Similarly, the C-OH/C-H ratio decreases from 4.979 in the control group to 2.75 in pH 13-60 °C-12 m, with a decrease of 44.8%. This observed decrease is inconsistent with theoretical expectations and may be attributed to two primary mechanisms. First, hydroxyl-containing hydrolysis products may leach from the material into the immersion solution, as evidenced by the gradual yellowing of the solution at higher pH levels observed during the experiments. Second, hydroxide ions (OH^−^) can react with Si-O-Si bonds in the basalt fibers, resulting in the formation of unstable Si-OH structures. These structures are subsequently converted into silicate ions (e.g., ${\mathrm{SiO}}_{3}^{2-}$), which then dissolve and migrate out of the material. This process leads to the loss of a portion of the newly generated hydroxyl groups, thereby contributing to their under-detection in the FTIR analysis.

#### 3.4.3. SEM

**Figure 10:**
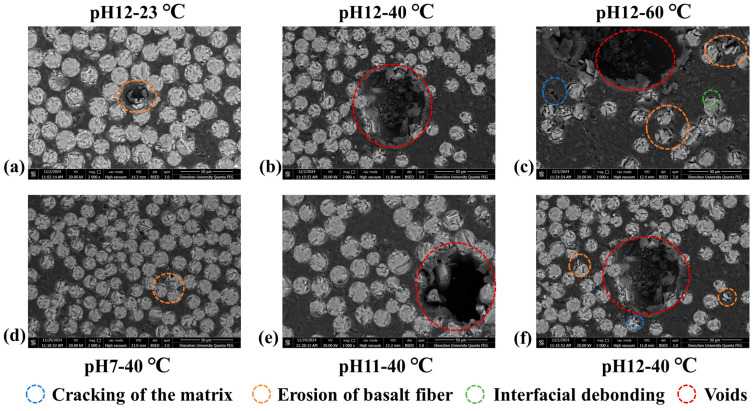
Micromorphology of conditioned BFRP rebars.

SEM images in Figure 10 indicate that pH and temperature markedly influence the microstructure of BFRP rebars. At 40 °C, neutral conditions (pH 7 in Figure 10d) preserve the microstructure: the resin matrix remains intact, fiber surfaces appear smooth, and the fiber–matrix interface is well bonded, with only occasional micro voids. Under mild alkalinity (pH 11 in Figure 10e), early degradation emerges, including fine matrix cracks, slight localized fiber-surface erosion, and limited interfacial debonding. At pH 12 (Figure 10b,f), deterioration intensifies, matrix cracking advances, fiber erosion becomes more pronounced, and interfacial debonding spreads over larger regions. These observations collectively show that increasing alkalinity exacerbates matrix cracking, fiber erosion, and interfacial debonding in BFRP rebars, with notably more severe effects in highly alkaline environments [16]. The absence of an SEM image for pH 13 is attributed to extensive fiber loosening under these conditions, which precluded specimen preparation for SEM imaging.

## 4. Degradation Mechanism

**Figure 11:**
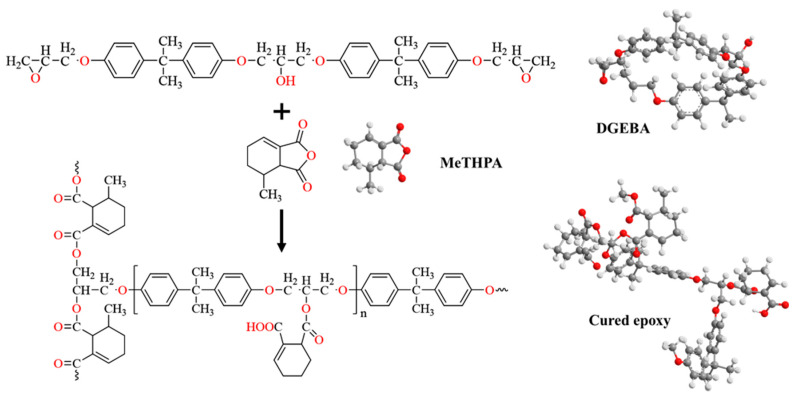
Schematic diagram of DGEBA epoxy resin curing.

The BFRP rebar in this study utilized a diglycidyl ether of bisphenol A (DGEBA)-based epoxy resin cured with methyl tetrahydrophthalic anhydride (MeTHPA) and catalyzed by 2,4,6-tris (dimethylaminomethyl) phenol (DMP-30) [33]. As depicted in Figure 11, curing yields a highly cross-linked three-dimensional polymer network primarily comprising ether linkages (C–O–C) and secondary hydroxyl groups [33]. Although the base resin cure does not intrinsically generate ester bonds, ester linkages can form via side reactions between the anhydride curing agent and residual hydroxyls. These structural motifs are thermally stable under ambient conditions but are chemically susceptible to alkaline attack [34].

In strongly alkaline environments (pH > 11), hydroxide ions (OH^−^) diffuse into the resin matrix and initiate nucleophilic attack on ester and ether linkages [12]. As outlined in Equations (9) and (10), ester groups are highly susceptible to alkaline hydrolysis and saponification, yielding alcohols and carboxylates, which induces chain scission and reduces molecular weight. Although ether bonds are comparatively more resistant, prolonged alkaline exposure, especially at elevated temperatures (e.g., 60 °C), can also cleave these linkages, further degrading the network and producing low-molecular-weight, viscous exudates at the surface.

Hydrolysis of ester linkages and consequent chain scission reduce cross-link density and promote matrix swelling; however, the glass transition temperature ($T_{g}$) remains relatively unchanged, indicating that the bulk thermal stability of the resin matrix is largely preserved despite localized degradation. This observation aligns with Rifai et al. [35], who reported stable $T_{g}$ values for BFRP bars conditioned in alkaline or moist concrete environments. Degradation byproducts manifest macroscopically as sticky surface films and voids, consistent with FTIR evidence showing attenuation or loss of the ester C=O band (1720–1740 cm^−1^) and the ether C–O–C band (1100–1150 cm^−1^). Comparable behavior has been documented by Shen et al. [36], who found that ester-containing biobased epoxies underwent rapid alkaline hydrolysis via surface erosion, whereas ether-rich DGEBA-based epoxies degraded more slowly.
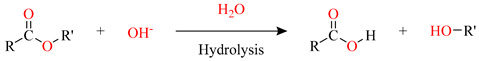
(9)
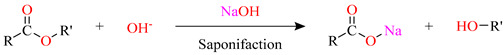
(10)

Water ingress contributes to epoxy degradation and interfacial weakening [37]. In alkaline environments, hydroxide ions (OH^−^) attack silane coupling agents, disrupting the chemical bridges that bond basalt fibers to the epoxy matrix [38]. Concurrently, degradation byproducts such as silanols (e.g., Si(OH)_4_) accumulate at the fiber-matrix interface, promoting local swelling and initiating microcracks that facilitate interfacial debonding and delamination. Consistent with this mechanism, Rao et al. [39] reported that OH^−^ cleaves Si–O bonds in silica, generating silanol-containing species analogous to those produced by silane degradation.

**Figure 12:**
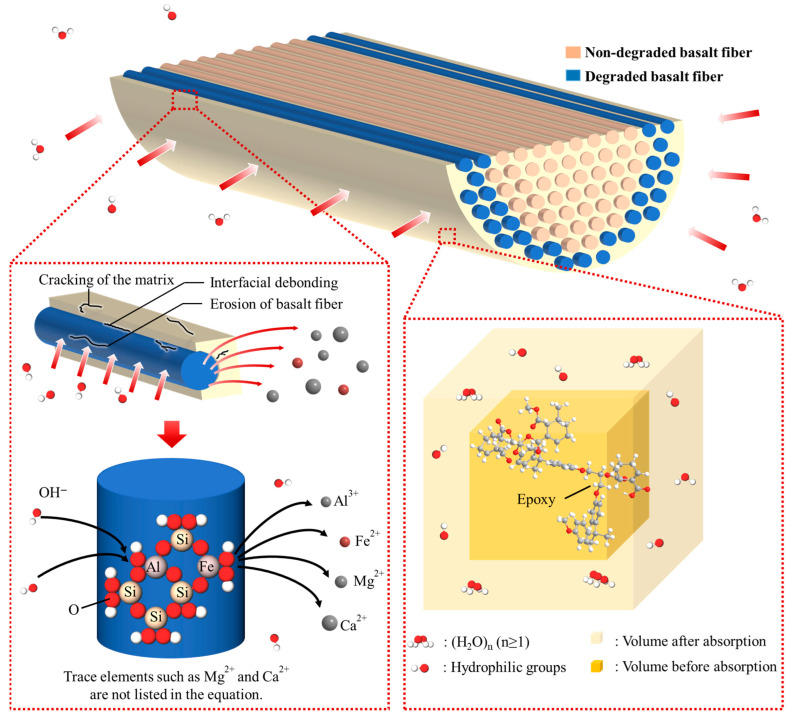
Degradation mechanism of BFRP rebars under alkali attack.

As illustrated in Figure 12, degradation initiates with matrix cracking and fiber surface erosion, followed by leaching of metal ions (e.g., Al^3+^ and Fe^2+^) from the basalt fibers into the surrounding medium. This ion exchange further accelerates deterioration of the fiber surface and compromises the interfacial bond. Simultaneously, the epoxy matrix absorbs water and swells, processes promoted by hydrophilic functional groups and elevated temperatures, thereby increasing the diffusivity of corrosive species [40].

The thermal expansion mismatch between fiber and resin, combined with internal stresses arising from moisture-induced swelling, further exacerbates interfacial degradation [38]. Over time, these coupled mechanisms facilitate deeper penetration of alkaline solutions and progressively undermine the composite’s structural integrity.

As the primary load-bearing phase in BFRP, basalt fibers undergo more severe degradation in alkaline environments, driven by chemical attack on their intrinsic constituents. The silicate network and associated metal oxides are susceptible to OH^−^ ingress, as evidenced by the key reactions outlined in [41]. In particular, the dominant Si–O–Si framework of basalt fibers is disrupted via nucleophilic attack by hydroxide ions.

≡Si–O–Si≡ + OH^−^ → ≡Si–OH + ≡Si–O^−^ (11)

≡Si–O^−^ + H_2_O → ≡Si–OH + OH^−^ (12)

These reactions initiate a self-propagating depolymerization cascade: cleavage of Si–O–Si bonds yields reactive silanol groups (≡Si–OH), which further catalyze network breakdown. In parallel, the release of Si–O^−^ species promotes ion-exchange processes (e.g., Ca^2+^ and Mg^2+^ within the fibers exchanging with Na^+^ from the alkaline medium), thereby destabilizing the fiber’s microstructure. Also, other metal oxides (e.g., Al_2_O_3_ and Fe_2_O_3_) may be dissolved in strong alkaline solutions [42]. Leaching of Al^3+^ and Fe^3+^ ions generates voids and defects within the fiber’s microstructure. The resultant dissolution perturbs the crystalline–amorphous phase balance, diminishing structural coherence.

Al_2_O_3_ + 2OH^−^ + 3H_2_O → 2[Al(OH)_4_]^−^ (13)

Fe_2_O_3_+ 2OH^−^ + 3H_2_O → 2[Fe(OH)_4_]^−^ (14)

These chemical attacks induce microstructure damage observable via SEM in Figure 10, such as matrix cracking, basalt fiber erosion, and interfacial debonding. The degradation of basalt fibers is a complex, multi-component, self-accelerating process governed by Equations (11)–(14). Both the silicate network (dominant phase) and metal oxides (stabilizing phases) play crucial roles in this process. The depolymerization of the silicate network initiates damage, while the dissolution of metal oxides exacerbates the formation of structural defects. Eventually, this leads to a collapse in strength, whereas the modulus (dependent on the integrity of the bulk network) demonstrates greater resilience.

These chemical and structural changes collectively stem from three primary degradation mechanisms that govern BFRP performance in alkaline environments. First, fiber corrosion, driven by OH^−^ attack on silicate networks and metal oxides in basalt fibers, leads to surface erosion and gradual dissolution. Second, resin matrix hydrolysis cleaves ester and ether bonds, reducing cross-link density and inducing swelling. Third, fiber-matrix interface debonding arises from chemical degradation of silane coupling agents and stress-induced microcracks from matrix swelling. These mechanisms interact synergistically: interface damage accelerates corrosive ion penetration, exacerbating both fiber corrosion and resin hydrolysis, which in turn further weaken the interface—creating a positive feedback loop that ultimately undermines load-bearing capacity.

These defects directly compromise the load-bearing capacity of the fiber and the efficiency of load transfer between the fiber and matrix, resulting in a degradation of mechanical properties. For tensile behavior, at pH = 13 and 60 °C, strength decreases by ~72% after 90 days as defects propagate under tension, causing premature fiber fracture. Notably, the modulus drops by only ~12%, a divergence arising because modulus depends on the bulk stiffness of the fiber network—short-term defects such as surface pits have limited impact on elastic response—whereas strength is governed by critical defect propagation exacerbated by chemical damage. This trend aligns with Wu et al. [15], who observed ~60% tensile strength retention after 9 weeks in a 55 °C alkaline solution. Flexural strength degrades rapidly under combined tension, compression, and shear, as matrix cracking couples with fiber damage (Equations (11)–(14)) to create delamination sites that disrupt load transfer across the composite. Interlaminar shear strength exhibits severe decline (76.6% loss at pH = 13, 60 °C, 90 days), consistent with Yi et al. [43], who reported a 57.78% reduction after 90 days in a pH = 13.2, 55 °C solution. Here, chemical damage weakens the fiber-matrix interface: degraded fiber surfaces (Equations (9) and (10)) reduce bonding with the epoxy matrix, making shear loads prone to inducing delamination.

## 5. Conclusions

In this study, the durability of BFRP rebars is systematically evaluated in distilled water (pH 7) and NaOH solutions of varying alkalinity (pH 11, 12, and 13). Based on surface morphology characterization, water absorption measurements, mechanical testing, and microstructural analysis, the following principal conclusions are obtained:

(1) The mechanical performance of BFRP deteriorates markedly under high temperature and strongly alkaline conditions. After exposure at pH 13 and 60 °C for 90 days, the retained tensile, flexural, and interlaminar shear strengths decrease by up to approximately 72%, 88%, and 77%, respectively, whereas the elastic moduli exhibit comparatively minor changes, indicating that strength is more sensitive to environmental degradation.
(2) Microscale characterization (SEM, DSC, and FTIR) clarifies BFRP’s alkaline degradation: SEM shows matrix cracking, fiber erosion, and interfacial debonding; FTIR detects resin ester/ether hydrolysis, but an abnormal C-OH/C-H drop implies product leaching only at the interface (via silane coupling agents); DSC’s stability $T_{g}$ rules out bulk matrix degradation. Together, they confirm resin chemical changes are interface-confined.
(3) Degradation of BFRP in alkaline media is governed by fiber corrosion, resin hydrolysis, and fiber-matrix interfacial debonding. Hydroxide ions attack Si–O–Si linkages in the fibers and ester groups in the matrix, thereby weakening both phases. Interfacial damage is further exacerbated by silane hydrolysis and crack initiation, with elevated temperature accelerating these processes. The resulting synergistic degradation promotes stress concentration and progressive failure. Notably, the glass transition temperature ($T_{g}$ ) of the resin matrix remains essentially unchanged, indicating that the bulk thermal stability of the epoxy network is preserved despite localized chemical and structural deterioration.
(4) In view of the identified degradation mechanisms and performance losses under combined alkaline and thermal exposure, the use of BFRP rebars is recommended in environments of mild alkalinity (pH ≤ 12) and service temperatures below 60 °C. Within these bounds, mechanical properties and thermal stability remain comparatively stable.

It should be noted that the short-term acceleration tests conducted in this study are performed under idealized alkaline solutions and controlled thermal conditions. These conditions do not fully replicate the complex physicochemical environments present in actual concrete structures. Factors such as cyclic loading, carbonation, and chloride/sulfur attack (particularly in seawater-sand concrete) are likely to play significant roles in BFRP degradation mechanisms, thereby limiting the direct extrapolation of our findings to real-world scenarios. This aspect should be addressed in future research. Nevertheless, despite these limitations, the study provides valuable insights into the long-term performance of BFRP rebars in low-alkaline environments (pH 11–12), offering essential technical support for the durability design of BFRP-reinforced concrete structures.

## Figures and Tables

**Table 1:** Physical and mechanical properties of BFRP rebars.

Item	Value	Code	Reference
Nominal radius, a (mm)	6 ± 0.1	ATSM D7205	[27]
Tensile strength, $\sigma_{TSL}$ (MPa)	771 ± 61.7	ATSM D7205	[27]
Flexural strength, $\sigma_{FLX}$ (MPa)	1071.8 ± 117.9	ASTM D4476M-14	[28]
Interlaminar shear strength, $\tau_{ILSS}$ (MPa)	17.9 ± 3.2	ASTM D4475-02	[29]

**Table 2:** Chemical composition of BFRP.

Component	SiO_2_	Al_2_O_3_	Fe_2_O_3_	CaO	MgO_2_	Na_2_O	K_2_O	TiO_2_	P_2_O_5_	MnO
Content (wt%)	51.506	16.963	11.871	8.386	5.175	2.628	1.846	1.258	0.226	0.141

**Table 3:** The diffusion coefficient of BFRP rebars.

Solution	Diffusion Coefficient (10^−7^ mm^2^/s)		
	23 °C	40 °C	60 °C
pH = 7	5.876	6.758	7.849
pH = 11	6.815	7.352	7.789
pH = 12	6.758	7.093	7.547
pH = 13	7.071	7.277	/

**Table 4:** FTIR results of BFRP rebars in terms of chemical bond ratio.

ID	O-H/C-H		C=O/C-H		C-O-C/C-H		C-OH/C-H	
	Value	Ratio	Value	Ratio	Value	Ratio	Value	Ratio
Control	0.208	-	2.507	-	4.364	-	4.979	-
pH 7-60 °C-12 m	0.22	+5.77%	2.32	−7.46%	3.521	−19.30%	3.567	−28.40%
pH 11-60 °C-12 m	0.346	+66.30%	2.1	−16.20%	3.331	−23.70%	3.485	−30.00%
pH 12-60 °C-12 m	0.368	+76.90%	1.856	−26.00%	2.946	−32.50%	3.067	−38.40%
pH 13-60 °C-12 m	1.015	+388%	0.743	−70.40%	2.173	−50.20%	2.75	−44.80%

## Data Availability

The original contributions presented in this study are included in the article. Further inquiries can be directed to the corresponding authors.
